# Cytotoxic Effects of Tropodithietic Acid on Mammalian Clonal Cell Lines of Neuronal and Glial Origin

**DOI:** 10.3390/md13127058

**Published:** 2015-11-27

**Authors:** Heidi Wichmann, Farina Vocke, Thorsten Brinkhoff, Meinhard Simon, Christiane Richter-Landsberg

**Affiliations:** 1 Aquatic Microbial Ecology Group, Institute for Chemistry and Biology of the Marine Environment (ICBM), University of Oldenburg, Oldenburg 26129, Germany; heidi.wichmann@uni-oldenburg.de (H.W.); thorsten.brinkhoff@icbm.de (T.B.); meinhard.simon@icbm.de (M.S.); 2 Biochemistry Group, Department of Neurosciences, University of Oldenburg, Oldenburg 26111, Germany; farina.vocke@uni-oldenburg.de; 3 Molecular Neurobiology, Department of Neurosciences, University of Oldenburg, Oldenburg 26111, Germany

**Keywords:** nerve cells, oligodendrocytes, cytoskeleton, mitochondria, oxidative stress, MAP kinases, *Roseobacter* clade bacteria

## Abstract

The marine metabolite tropodithietic acid (TDA), produced by several *Roseobacter* clade bacteria, is known for its broad antimicrobial activity. TDA is of interest not only as a probiotic in aquaculture, but also because it might be of use as an antibacterial agent in non-marine or non-aquatic environments, and thus the potentially cytotoxic influences on eukaryotic cells need to be evaluated. The present study was undertaken to investigate its effects on cells of the mammalian nervous system, *i.e.*, neuronal N2a cells and OLN-93 cells as model systems for nerve cells and glia. The data show that in both cell lines TDA exerted morphological changes and cytotoxic effects at a concentration of 0.3–0.5 µg/mL (1.4–2.4 µM). Furthermore, TDA caused a breakdown of the mitochondrial membrane potential, the activation of extracellular signal-regulated kinases ERK1/2, and the induction of the small heat shock protein HSP32/HO-1, which is considered as a sensor of oxidative stress. The cytotoxic effects were accompanied by an increase in intracellular Ca^2+^-levels, the disturbance of the microtubule network, and the reorganization of the microfilament system. Hence, mammalian cells are a sensitive target for the action of TDA and react by the activation of a stress response resulting in cell death.

## 1. Introduction

The marine environment harbors a variety of organisms producing a high diversity of structurally unique natural products. These substances show a broad spectrum of biological activities, including anti-cancer, antimicrobial, antifungal, antifouling effects and some are even able to alter mammalian neurological activity [1,2,3]. Marine bacteria represent a promising resource for novel secondary metabolites with largely undescribed targets, including the central nervous system (CNS). A plethora of marine bacteria are known to produce biologically active compounds, such as members of the *Roseobacter* clade [4,5,6]. The *Roseobacter* clade belongs to *Alphaproteobacteria* and represents a major fraction of bacterial communities in different marine habitats [7,8,9]. The production of diverse secondary metabolites may be one reason for the common occurrence and abundance of these bacteria in different habitats, for instance, by providing a competitive benefit over other species [6,10].

Tropodithietic acid (TDA), first described in 2004 [6,11], is produced by several organisms affiliated with the genera *Phaeobacter*, *Ruegeria*, and *Pseudovibrio* [12], all belonging to the family *Rhodobacteraceae*. TDA inhibits a broad spectrum of both Gram-positive and -negative bacteria, including clinical pathogens, fungi and microalgae [6,11,13,14], but no toxicity against the multicellular eukaryotic model organisms *Artemia* sp. and *Caenorhabditis elegans* was observed [15]. Due to TDA production, *Phaeobacter* and *Ruegeria* spp. were considered as potential probiotic organisms in aquacultures [16], however, not much is known about the interaction of TDA with eukaryotic cells and its effects on neural cell lines so far have not been investigated.

The purpose of the present study was to investigate the effects of TDA on cells of the mammalian nervous system. Towards this two cell lines representing the main types of the nervous system, *i.e.*, nerve cells and glia, were used, namely mouse neuroblastoma N2a cells as a model for neuronal cells [17] and rat oligodendroglial OLN-93 cells as a model for the myelin forming cells of the CNS [18]. The effects of TDA on cell viability and cell morphology were evaluated. Furthermore, mitochondrial integrity and the impact on Ca^2+^ influx were determined.

## 2. Results and Discussion

### 2.1. TDA Induced Cytotoxicity, Upregulation of Hsp32 and ERK1/2 Activation

**Figure 1:**
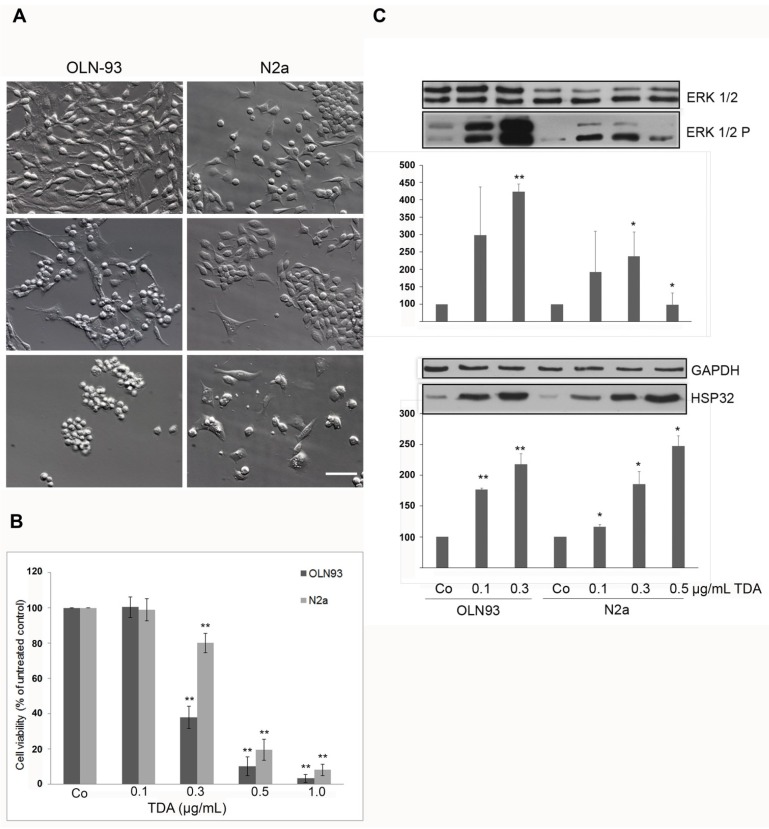
Cytotoxic effects of tropodithietic acid (TDA) in OLN-93 and N2a cells after incubation with tropodithietic acid (TDA) for 24 h. (**A**) Effect of TDA on cell morphology. Hoffman modulation contrast images are shown. OLN-93 and N2a cells were either treated with dimethyl sulfoxide as negative control (Co) or subjected to 0.3 µg/mL (1.4 µM) or 0.5 µg/mL (2.4 µM) TDA. Scale bar 50 µm. (**B**) MTT (thiazolyl blue tetrazolium bromide) assay. Cells were incubated with increasing TDA concentrations (0.1–1 µg/mL, as indicated) (OLN-93 dark grey bars, N2a cells light grey bars). (**C**) Western blot analysis. Cell lysates of OLN-93 and N2a cells were prepared and subjected to immunoblot analysis using antibodies as indicated on the right (for details see Experimental Section). Quantitative evaluation of the immunoblots was carried out by densitometric scanning and Image Quant software (Molecular Dynamics, Sunnyvale, CA, USA). Activated ERK 1/2 (ERK 1/2 P) is expressed as percentage of the total amount of ERK1/2 (100%). Hsp32 is expressed as percentage of glyceraldehyde 3-phosphate dehydrogenase (GAPDH, 100%), which was used as loading control (100%). Figure 1B,C: Statistical evaluation was carried out by students *t*-test: * *p* < 0.05 significant and ** *p* < 0.001 highly significant compared to the control.

To investigate the cytotoxic potential of TDA cells were incubated for 24 h with increasing concentrations. Figure 1A shows that in both cell lines morphological changes occurred at 0.3 µg/mL (1.4 µM), cell density was decreased and cellular processes were retracted. At 0.5 µg/mL (2.4 µM), severe cytotoxic effects were observed (Figure 1A).

To quantitatively assess the cytotoxic effects, an MTT (thiazolyl blue tetrazolium bromide) viability assay was carried out, which revealed that at a concentration of 0.3 µg/mL viability was decreased to 40% in oligodendroglial OLN-93 cells and to 80% in neuronal N2a cells (Figure 1B). After 24 h incubation with 0.5 µg/mL of TDA, only 10% of OLN-93 cells and 20% of N2a cells were viable, and the treatment with 1.0 µg/mL resulted in cell death of more than 90% of the cells (Figure 1B). Hence, OLN-93 cells were more sensitive to the treatment than N2a cells, and in both cell lines TDA exerted cytotoxic effects at a much lower concentration than previously observed in MCF7 cells (breast carcinoma), HM02 cells (gastric carcinoma) and HEPG2 cells (hepatocellular carcinoma). In these cells, cytotoxicity occurred at concentrations above 8 µg/mL [11]. This indicates that neuronal and glial cells display a higher sensitivity to TDA than cell lines derived from other organs than the brain.

To further study the molecular events underlying the cytotoxic effects of TDA, immunoblot analysis was carried out. Since OLN-93 cells displayed a higher sensitivity and cells started to round up, clump and detach from the substrate at a TDA concentration of 0.5 µg/mL (Figure 1A,B), this concentration was omitted and immunoblots were prepared only from cells incubated with 0.1 and 0.3 µg/mL of TDA. OLN-93 and N2a cells were incubated for 24 h with TDA as indicated, cell lysates were prepared and the activation of the extracellular signal-regulated kinases 1 and 2 (ERK1/2), and the induction of heat shock protein 32 (HSP32) or so-called heme oxygenase 1 (HO-1) was investigated. ERK1/2, which belongs to the family of MAP kinases, has been connected to protective mechanisms in a number of studies, but also death promoting activity is likely [19]. HO-1 interacts with the MAPK cascade and is involved in the regulation of oxidative stress [20,21]. Immunoblot analyses using antibodies recognizing the dually phosphorylated forms of ERK1/2 (ERK1/2P), antibodies directed against total ERK1/2, and antibodies against HSP32 were carried out. GAPDH was used as a loading control. Figure 1C demonstrates that TDA at 0.1–0.3 µg/mL caused the activation of ERK1/2 with no concomitant changes in the amount of total ERK1/2 in both cell lines and that the effect was more prominent in OLN-93 cells than in N2a cells. In addition, a concentration dependent induction of HSP32 was observable, which was similar in both cell lines (Figure 1C). Thus, treatment with TDA exerted a stress response in neural cells. Upregulation of HSP32/HO-1 initially after the stress is considered a protective means, but at later chronic stages has pathological consequences. As shown before, OLN-93 cells and primary oligodendrocytes respond to oxidative stress exerted by hydrogen peroxide by upregulation of HSP32/HO-1, the activation of ERK1/2, and the onset of programmed cell death [21,22]. HSP32/HO-1 may be considered as sensor of oxidative stress. The stress regulated kinases ERK1/2 have been shown to be activated under similar conditions that induce HO-1 transcription [23], and it has been suggested that the rapid and transient activation of ERK1/2 enhances the survival capabilities of cells, while a delayed response participates in the regulation of cell death [22,24,25]. The present data indicate that in neural cells the cytotoxic effects of TDA are associated with the induction of oxidative stress.

**Figure 2:**
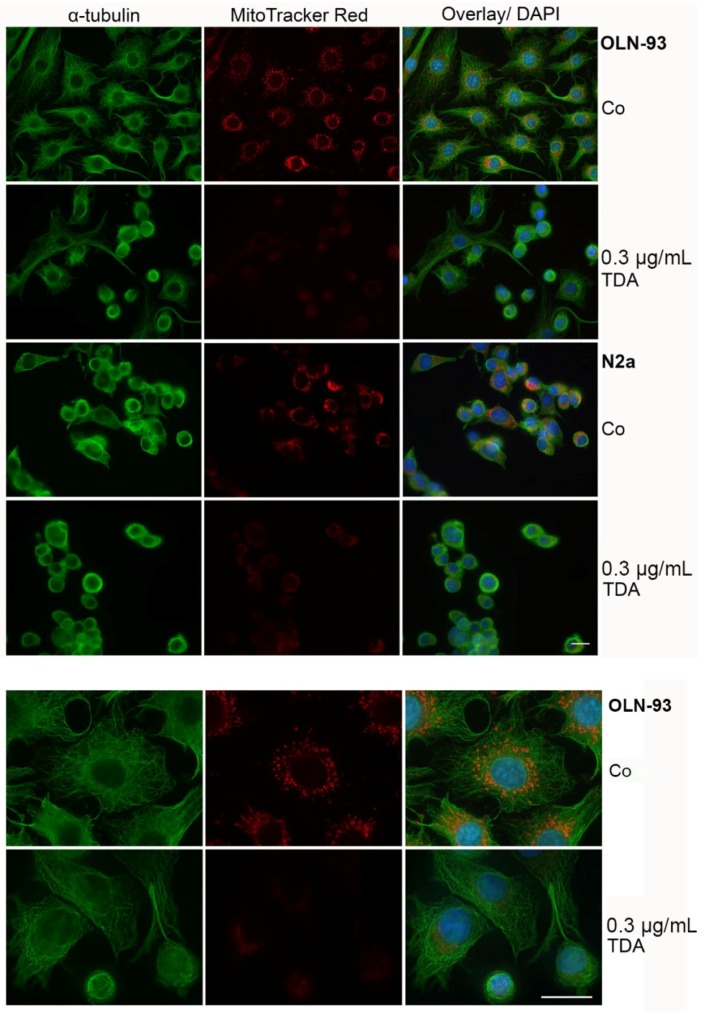
Effect of TDA on microtubule organization and mitochondrial integrity. Cells were subjected to 0.3 µg/mL TDA for 24 h. Subsequently, cells were incubated for 30 min with MitoTrackerRed, fixed with methanol and subjected to indirect immunofluorescence using antibodies against α-tubulin (green). Nuclei were stained with DAPI (blue). Co, untreated control incubated with the solvent DMSO only. Lower panel, OLN-93 cells shown at a higher magnification. Scale bars: 20 µm.

### 2.2. TDA Affects Microtubule Organization and Causes Mitochondrial Impairment

**Figure 3:**
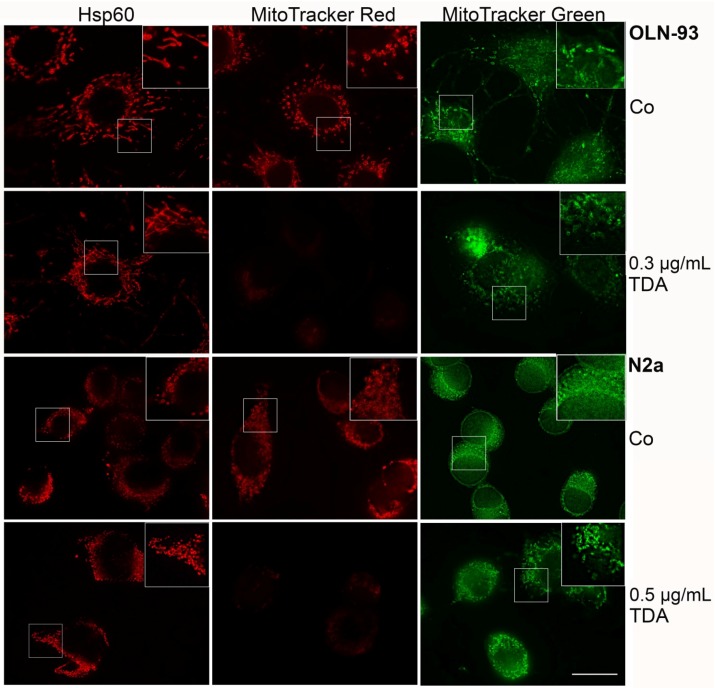
Effect of TDA on mitochondrial integrity. Cells were subjected to TDA for 24 h as indicated. Thereafter, cells were subjected to indirect immunofluorescence using antibodies against heat shock protein 60 (Hsp60), or incubated for 30 min with MitoTrackerRed or MitoTrackerGreen. Co, untreated control incubated with the solvent DMSO only. Scale bar: 20 µm.

To test whether morphological changes and cell death are causally related to the impairment of microtubule organization and mitochondrial damage, mitochondria were stained with MitoTrackerRed, followed by indirect immunofluorescence using antibodies against α-tubulin. MitoTrackerRed binds only to intact mitochondria and is used to assess the integrity of the membrane potential. Thus, the fluorescent signal is more prominent in healthy mitochondria. Cells were incubated with TDA for 24 h as indicated and then analyzed. Figure 2 demonstrates that in both cell lines the microtubule network is disorganized and a decrease in mitochondrial staining was observed, indicating a loss of the mitochondrial membrane potential. To further assess mitochondrial integrity and distribution, cells treated under the same conditions were labeled with MitoTrackerGreen, which is taken up by mitochondria regardless of their functional state, and indirect immunofluorescence was carried out with antibodies against heat shock protein 60 (Hsp60). Hsp60 is constitutively expressed in the cells and localized in the mitochondria. It is associated with the mitochondrial matrix and involved in the folding and assembly of transported protein into the mitochondrium [26,27]. As depicted in Figure 3, after treatment with TDA mitochondria appeared smaller and more condensed, however, although the membrane potential was impaired, they remained distributed throughout the cytoplasm and within the cellular processes. Furthermore, Hsp60 remained associated with the mitochondria and was not released into the cytoplasm.

Oxidative stress, a variety of chemicals and Ca^2+^ influx have been connected to mitochondrial pore opening, which causes mitochondrial membrane depolarization and thus may lead to necrotic or apoptotic cell death [28,29,30]. Our data indicate that TDA similarly leads to a breakdown of the mitochondrial membrane potential, yet without having an impact on mitochondrial distribution. Under the present conditions, necrotic rather than apoptotic cell death occurred, since cellular nuclei were not pyknotic and no DNA fragmentation was detectable (data not shown). It may be possible that the extent of damage was too severe and the cells die due to inadequate energy production [31]. Taken together, mitochondrial and cytoskeletal alterations are involved in the cytotoxic effects exerted by TDA.

### 2.3. Intracellular Ca^2+^-Levels Are Increased after Supplementing TDA

**Figure 4:**
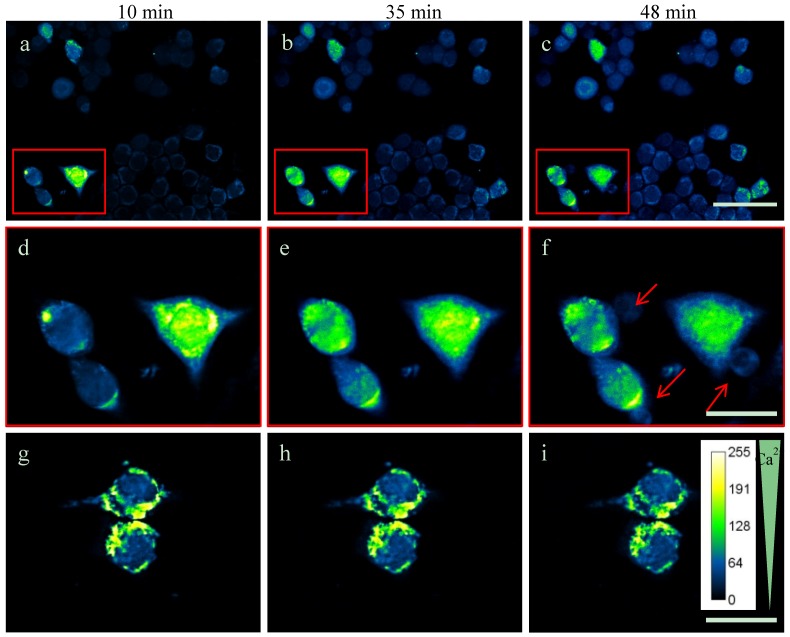
Fura-2 AM calcium imaging. TDA induces an increase of intracellular Ca^2+^ in N2a cells. (**a**–**c**) Cells were incubated with Fura-2-AM solution at 37 °C in the dark. Subsequently, 0.1 µg/mL TDA was added and cells were monitored after 10 min, 35 min and 48 min, as indicated. Scale bar: 50 µm. (**d**–**f**) Enlargement of the insets depicted in the upper panels (red arrows: necrotic cells). Scale bars: 20 µm. (**g**–**i**) Negative control without TDA additive. Scale bars: 20 µm.

A disturbance of the intracellular calcium homeostasis contributes to mitochondrial damage, cytoskeletal disorganization and cell death [32]. To explore whether TDA may influence the Ca^2+^ concentration within the cells, we measured the intracellular Ca^2+^ concentration using Fura-2 AM calcium imaging in N2a cells (Figure 4). Since these experiments were carried out at isotonic conditions and at a rather low cell density, 0.1 µg/mL TDA was used to monitor the effects. At higher concentrations (0.3– 0.5 µg/mL), the toxic effects were rather severe and the changes in intracellular Ca^2+^ levels were difficult to demonstrate.

The Fura-2 AM calcium imaging was recorded on video and representative images are shown (Figure 4). The amount of cells with augmented Ca_i_^2+^ concentration increased over time, cell swelling occurred and cell lysis was observed after 48 min (Figure 4f, red arrows).

A number of chemical substances and toxic agents are known to cause an imbalance of Ca^2+^ homeostasis and a lethal influx of Ca^2+^ into cells [29,32]. Ca^2+^ is a mediator of necrosis, whose core event is bioenergetic failure and rapid loss of plasma membrane integrity [33]. In addition, oxidative stress can promote cell death and mitochondria are sensitive targets. Induction of mitochondrial permeability transition (MPT), which results in opening large conductance permeability transition pores and makes the inner membrane permeable, allows Ca^2+^ to leave the mitochondrion. Additionally, depolarization of the Δψm results in an uncontrolled flow of protons and some molecules across the outer membrane. Recently, investigations raised the possibility that mitochondria might act as large and dynamic physiological Ca^2+^ buffer [29,30]. The exact molecular mechanisms underlying TDA induced cell death need to be further investigated.

### 2.4. Influence of TDA on Microfilament Organization

**Figure 5:**
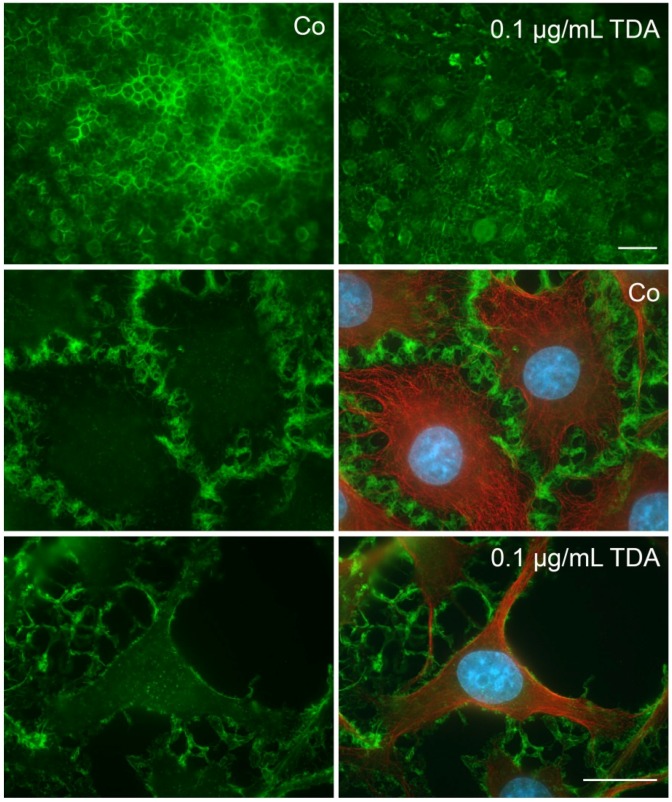
Effect of TDA on F-actin cytoskeleton in OLN-93 cells. Cells were subjected to 0.1 µg/mL TDA for 24 h. Subsequently, cells were fixed with 3% paraformaldehyde, incubated with phalloidin green and indirect immunofluorescence using antibodies against α-tubulin (red) was carried out. Nuclei were stained with DAPI (blue). Co, untreated control. Scale bars: 50 µm (upper two images, 200× magnification), 20 µm (1000× magnification).

Local cell adhesion and attachment to the extracellular matrix is connected to local Ca^2+^ signals, and the cytoskeleton, in particular the sensitive microfilament system, reacts to oxidative stress and elevation of free Ca^2+^ [34]. To study the effect of TDA on microfilament organization, OLN-93 cells were treated with 0.1 µg/mL TDA for 24 h, and filamentous actin (F-actin) was stained by phalloidin-green. Figure 5 demonstrates that TDA severely affected F-actin organization.

While in control cells an actin rich network is prominently expressed and clearly observed at the outer boundaries of the cells, in TDA treated cells it appears more diffuse, diminished and retracted. This might be causally related to the observed changes in the intracellular Ca^2+^ concentration and/or to a possible oxidative insult exerted by TDA. ATP deprivation, potentially induced by TDA, due to mitochondrial Ca^2+^ overload, the loss of Δψm and mitochondrial impairment, or other indirect or direct mechanisms, may cause dramatic actin cytoskeletal reorganization [31,35,36]. A similar unusual actin relocalization was described in a study by Hrouzek *et al.* [35]. These authors showed that cyanobacterial derived compounds induced necrosis in HeLa cells via membrane permeabilization and an increase of Ca^2+^-influx. In addition, the involvement of cell adhesion molecules is rather likely. 

The present data indicate that the microfilament network is a sensitive target for TDA induced cytotoxicity. However, further studies are needed to elucidate the molecular mechanisms and signal transduction pathways underlying these effects. This is in particular interesting in the context of energy metabolism and a possible influence on cell adhesion processes.

## 3. Experimental Section

### 3.1. Materials and Antibodies

Cell culture media were purchased from Gibco/BRL (Grand Island, NY, USA). Poly-l-lysine (PLL), from Sigma (Munich, Germany). Tropodithietic acid (TDA) was purchased from BioViotica Naturstoffe GmbH (37077 Göttingen, Germany) dissolved in DMSO and stored in dark at −20 °C. Initial studies were carried out using methanol as a solvent, since DMSO might bind to TDA and alter its activity [11]. In comparison to DMSO as a solvent, a similar cytotoxicity was observed, however, TDA in methanol solution showed a tendency to form aggregates. Hence DMSO was used and always included in control experiments.

For Western blot analysis, the following antibodies were used, working dilutions are given in brackets: mouse monoclonal antibody (mAb), anti-α-tubulin (1:1000), anti-extracellular regulated kinase 1,2 (ERK1,2; 1:2000), and mouse mAb ERK1,2-P (1:1000) from Sigma (Munich, Germany); mouse mAb anti-GAPDH (1:1000) from Sigma-Aldrich (St. Louis, MO, USA); and monoclonal antibodyanti-HSP32 (1:1000) from Enzo Lifesciences (Lörrach, Germany).

### 3.2. Cell Culture

In this study, OLN-93 cells, an oligodendroglial cell line derived from rat brain glial cultures [18] and N2a (wt) cells, a mouse derived neuroblastoma cell line [17], were used. Cells were kept in Dulbecco’s modified Eagle medium (DMEM) supplemented with 10% heat inactivated fetal bovine serum (FBS) for OLN-93 cells and 0.5% FBS for N2a cells, 2 mM glutamine, 50 U/mL penicillin (P), and 50 μg/mL streptomycin (S) at 37 °C and 10% CO_2_ [18]. In all subsequent experiments, DMSO was added to control cultures. All experiments were carried out at least three times with similar results. Cells were monitored by Hoffman modulation contrast microscopy.

### 3.3. Immunoblot Analysis

Cellular monolayers of control and treated cells were washed once with PBS, scraped off in sample buffer containing 1% SDS, and boiled for 10 min. The protein contents were determined according to Neuhoff *et al.* [37]. For immunoblotting, total cellular extracts (10–20 μg protein per lane) were separated by one-dimensional SDS polyacrylamide gel electrophoresis (SDS-PAGE) using 8.75%–10% polyacrylamide gels and blotted onto nitrocellulose membranes (Whatman, Dassel, Germany; 0.2 μm). The blots were saturated with TBS (20 mM Tris, 136.8 mM NaCl, pH 7.5) containing 5% dry milk and incubated with the individual antibodies overnight at 4 °C. After washing with Tris-buffered saline (TBS) with 0.1% *v*/*v* Tween 20 (TBS-T), blots were incubated with HRP-conjugated anti-mouse (1:10000) or anti-rabbit (1:10000) antibodies for 1 h at RT. After washing with TBS-T, blots were visualized by the enhanced chemiluminescence procedure as described by the manufacturer (Thermo Scientific, Rockford, IL, USA). All experiments were carried out at least three times with similar results.

### 3.4. Mitochondrial Staining

OLN-93 cells were grown on PLL-coated glass cover slips, N2a cells were grown without PLL and incubated with MitoTracker Red (100 nM) or MitoTracker Green (150 nM) (Molecular Probes, Oregon, OR, USA) for 30 min, washed twice with PBS and fixed with ice-cold methanol for 7 min (MitoTracker Green) or with 3% paraformaldehyde (MitoTracker Red). The latter were permeabilized with 0.1% Triton X-100. Thereafter, indirect immunofluorescence staining was carried out as described below.

### 3.5. Indirect Immunofluorescence

OLN-93 cells (3.5 × 10^5^ cells/10 cm dish) were cultured on PLL-coated glass coverslips for 24 h in DMEM/1% FBS and subjected to treatment as indicated. N2a cells (1.2 × 10^6^ cells/10 cm dish) were cultured on HCl treated glass coverslips without PLL for 24 h in DMEM/0.5% FBS. Cells were incubated overnight at 4 °C with the following antibodies (the working dilutions are given in brackets): mouse mAb anti-α-tubulin (1:250) (Sigma, Munich, Germany). Actin Staining was performed with Phalloidin labeled with FITC (Phalloidin Green, 1 µM) from Sigma (Munich, Germany) and mouse mAb anti-HSP60 (1:1000) from Enzo Life Sciences. After washing with PBS, cells were incubated for 1 h with Dylight (488 or 594) conjugated (1:400; Thermo Scientific, Rockford, IL, USA) and FITC-conjugated (1:100) secondary antibodies (Santa Cruz Biotechnology, Inc., Heidelberg, Germany) washed with PBS and mounted. Nuclei were stained by 4,6-diamidino-2-phenylindole (DAPI) (1.5 mg/mL) included in the mounting medium (Vectashield; Vector Laboratories, Burlingame, CA, USA). Fluorescent labeling was studied using a Zeiss epifluorescence microscope (Oberkochen, Germany) equipped with a digital camera using a plan neofluar objective (40× magnification for overview images, 100× magnification for detailed images).

### 3.6. MTT-Viability Assay

To assess the cytotoxic potential of TDA the MTT (tetrazolium) assay was carried out as described before [38]. Briefly, OLN93 or N2a cells were prepared as described above, plated on (PLL-coated) 96-microwell cell culture plates (3500 or 12,000 cells per well) and incubated for 24 h. The growth medium was removed and fresh medium (100 µL/well) was added. Cells were then incubated with 0.1–1 µg/mL TDA and incubated for 24 h. Ten microliters of MTT solution (5 mg/mL in PBS) were added to the wells (each containing 100 µL medium) and the plates were incubated for 2 h. One hundred microliters of a solubilization solution (10% sodium dodecyl sulfate in 0.01 mol/L HCl) was added and incubated overnight to dissolve the water-insoluble formazan salt. Quantification was carried out with an ELISA reader at 595 nm using a 655-nm filter as a reference. Data are expressed as percentage of the untreated controls, with each value representing the mean SD of eight microwells from three independent experiments (*n* = 24).

### 3.7. Fura-2 AM Staining

The procedure was carried out as described by the manufacturer (Molecular Probes, Oregon, OR, USA). Briefly, chamber slides were coated with 0.1 mg/mL poly-l-lysine. After washing with PBS, cells (about 1 × 10^5^/well) were incubated in DMEM/10% FCS for 2 days at 37 °C and 5% CO_2_. The cells were washed two times with PBS and incubated for 1 h in 5 µM Fura-2-AM solution at 37 °C in the dark. Within the cells, the Fura-2-Acetoxymethylester is cleaved by cellular esterase by which Fura-2 is activated and trapped in the cell. It binds free Ca^2+^ ions and Fura-2-AM-Ca^2+^ complex can be detected by fluorescence microscopy. Two additional washing steps followed and cells were further incubated for 30 min in PBS at 37 °C in the dark to allow the reaction to complete. For fluorescence imaging, the cells were washed again with PBS and covered with 200 µL PBS and focused under a fluorescence microscope (Olympus IX81; LUCP PlanFi 40×/0.60 objective, Hamburg, Germany). The fluorescence emission was detected every minute at 510 nm by an excitation of 340 and 380 nm for 10 min. Thereafter, 0.1 µg/mL TDA was added, and fluorescence was documented for further 60 min. The evaluation was carried out with the xcellence software (Olympus) as well as the freeware program Fiji Image J.

## 4. Conclusions

Previous studies suggested that TDA is non-hazardous to multicellular eukaryotic model organisms [15]. Here we showed, however, that TDA can have severe cytotoxic effects on mammalian neuronal and oligodendroglial cell lines in culture. These effects include mitochondrial damage, disorganization of the cytoskeleton, *i.e.*, the microtubule and microfilament network, an influx of intracellular calcium, and the upregulation of the heat shock protein Hsp32/HO-1 and the stress activated MAP kinases ERK1/2. Hence, cells respond to the treatment with TDA by activation of a stress response, which cannot counteract the cytotoxic effects and protect the cells. These cytotoxic effects occur at concentrations of 0.3–0.5 µg/mL (1.4–2.4 µM) TDA. To date, no data of natural concentrations of TDA in environmental samples exist, though in pure cultures of TDA producing bacterial strains the maximum concentration can be up to 450 µM [12]. Cell densities of TDA producers as found in these pure cultures are, however, unlikely in natural habitats and dilution effects in the marine environment would probably not allow TDA concentrations that caused a toxic effect in our experiments. Previous studies with fish and shellfish larvae showed indeed that the use of TDA-producing bacteria as probiotics in aquacultures was beneficial for larval development [16]. The minimum inhibitory concentration (MIC) of TDA for various bacteria, however, is at least hundredfold higher [16] than the concentration at which we observed cytotoxic effects. Our results therefore indicate that application of TDA in high concentrations as a pure substance should be treated with caution.
